# Plant Polyphenols and Human Health: Novel Findings for Future Therapeutic Developments

**DOI:** 10.3390/nu15173764

**Published:** 2023-08-28

**Authors:** Antonella Di Sotto, Silvia Di Giacomo

**Affiliations:** 1 Department of Physiology and Pharmacology “V. Erspamer”, Sapienza University of Rome, P.le Aldo Moro 5, 00185 Rome, Italy; 2 Department of Food Safety, Nutrition and Veterinary Public Health, Istituto Superiore di Sanità, 00161 Rome, Italy

Polyphenols are a large class of plant secondary metabolites that are biosynthetically derived from the shikimate pathway and characterized by aromatic rings with one or more hydroxyl groups [1,2,3]. Flavonoids, tannins, stilbenes, and lignans are the major polyphenolic groups; moreover, other phenolic compounds, namely phenolic acids (e.g., gallic acid, caffeic acid, and cinnamic acid), which are produced by the same pathway, are included in this class [1,2,3]. These substances are widely distributed in a variety of fruits, vegetables, grains, nuts, seeds, and beverages like tea, coffee, and wine and contribute to their organoleptic properties (e.g., bitterness, astringency, and flavor) and stability [1,2,3]. Their production in plants is tightly regulated by the plant’s interactions with its environment, and they are especially involved in plant defense against pathogens and harmful factors [4].

Plant polyphenols have received a significant scientific interest over the years owing to their promising healing properties, especially their antioxidant and anti-inflammatory effects [1,5,6]. Indeed, epidemiological evidence highlighted the ability of food polyphenols to boost human health and prevent several diseases, including cardiovascular, neurological, and liver diseases, in addition to diabetes, atherosclerosis, aging, and cancer [1,7,8]. Recent evidence has also highlighted the ability of some phenolics to counteract viral infections and to modulate the gut microbiota, thus favoring the host’s general health [9,10,11]. 

The Mediterranean diet (MD) is a special source of polyphenols, as it is based on an intake of vegetables, fruits, olive oil, and nuts, despite also involving the moderate consumption of alcohol, red and processed meat, butter, and sugary drinks [12]. Over the years, several studies have highlighted the beneficial effect of the MD in reducing the incidence of several diseases, including metabolic syndrome, neurodegeneration, and cancer [12]. Instead, the opposite effects, with increased risks of hypertension, glucose intolerance, abdominal obesity, and hyperlipidemia, have been shown in people consuming high levels of fat, sugars, and animal proteins [13,14].

Therefore, polyphenol supplementation via diet can represent a promising pharmaceutical and nutraceutical strategy. However, some pharmacokinetic issues, including reduced intestinal absorption and bioavailability and rapid metabolic modifications, seem to represent the main limits in harnessing the remarkable properties of polyphenols [15].

This Special Issue was aimed at gathering novel findings about plant polyphenols and phenolic-based extracts to enhance the available knowledge about the mechanisms underlying the properties of these natural substances, the pharmaceutical formulations developed to improve their bioavailability, and the clinical evidence in support of their use.

Some studies focused on the benefits of a polyphenol-based diet for an individual’s well-being; in particular, Aiello et al. [16] studied the metabolic status of Italian and Spanish university students with respect to fruit and vegetable consumption, highlighting a positive impact of vegetables, in addition to physical activity, on the students’ body composition profiles and glucose concentrations. Interestingly, another study highlighted that polyphenol supplementation during pregnancy can represent a suitable strategy for counteracting the risk of postnatal metabolic disorders associated with high-fat maternal diets or obesity [17]. Increasing evidence also showed that polyphenol supplementation through maternal diet, particularly resveratrol and its derivatives, can exert protective effects towards the damages induced by neonatal hypoxia–ischemia [18].

A particular topic addressed in this Special Issue is the role of polyphenolic compounds in vascular diseases, which occur widely in people of different ages; early signs can manifest even in healthy subjects and, if left untreated, can progress to symptomatic cardiovascular conditions [19]. Several studies highlighted that polyphenol-rich diets provide protective effects against cardiovascular diseases by virtue of the antioxidant, anti-inflammatory, antiplatelet, and vasodilatory properties of the polyphenols [20]. 

In this context, Martelli et al. [21] highlighted the potential benefits of the grape pomace polyphenolic extract Taurisolo^®^, which was microencapsulated with maltodextrins, on the integrity and functionality of the vascular endothelium in different experimental models; they also demonstrating the involvement of key pathways. These benefits were confirmed in clinical tests of endothelial reactivity. Similarly, Kudo et al. [22] studied two tannin-based preparations from the Japanese persimmon (*Diospyros kaki* L.), i.e., the juice from the immature fruit and the leaf powder dissolved in water, and highlighted that the samples possessed vasorelaxant properties and were able to inhibit vasocontraction via the hyperpolarization of smooth muscle cells. Instead, Dias et al. [23] showed that 3-hydroxyphenylacetic (3-HPAA), a quercetin metabolite produced by the human gut microbiota, induced relaxation in blood vessels and then modulated blood pressure in vivo.

Other Authors explored the anticancer powers of plant polyphenols in an attempt to develop possible preventive strategies against cancer; however, the clinical interest remains to be elucidated [24]. In this context, oleuropein, a peculiar phenolic compound from an olive leaf extract, exhibited antiproliferative and antimigration properties in an in vitro model of testicular cancer [25]; likewise, in vitro anticancer properties were highlighted for an extract from the fresh rhizomes of *Rhodiola rosea* L., which mainly contain free flavonoids and polyphenols [26]. Finally, nanoparticles of piceatannol, a more stable metabolite of the natural stilbene resveratrol produced by cytochrome P450 enzymes in humans, were able to prevent estradiol-induced endometrial hyperplasia in vivo, a condition which may increase the risk of endometrial malignancy [27]. In line with this evidence, a combination of resveratrol and viniferin produced hepatoprotective effects in a rat model of severe acute liver failure, suggesting an interest in stilbene combinations as nutraceutical strategies for preventing liver injury [28]. Moreover, Ruiz-Hurtado et al. [29] remarked on a potential gastroprotective power of propolis, a resinous substance produced by honeybees which is a source of diverse phenolic compounds, particularly flavonoids and phenolic acids. At both preclinical and clinical levels, promising benefits have been also highlighted for the oral supplementation of green tea polyphenols in skin ailments, especially with respect to photoprotection, which are likely due to their promising antioxidant, anti-inflammatory, and immunomodulatory effects. However, some issues regarding the clinical efficacy and safety profiles of these preparations remain to be clarified, thus encouraging further studies on the topic [30].

Finally, an exciting topic addressed in this Special Issue is the neuroprotection offered by plant polyphenols. Emerging evidence indicates an increase in brain-related disorders, likely associated with the aging of the population and environmental pollution, despite limited therapeutic options [8]. This strengthens the need to discover alternative strategies aimed at preserving brain health and preventing neurodegeneration and the progressive loss of neurons. In this context, polyphenol-based nutraceuticals have been highlighted over the years as interesting candidates for counteracting chronic degenerative disease in the central nervous system, as they are endowed with antioxidant and anti-inflammatory properties and are able to favor the balance of intestinal microbiota, thus promoting the health of the gut and brain [8].

In line with this evidence, Wdowiak et al. [31] described the pro-health activities of hesperidin and hesperetin, peculiar polyphenols of citrus fruits. Hesperidin was especially attractive for counteracting chronic diseases, such as neurodegenerative diseases and diabetes, owing to its antioxidant and anti-inflammatory powers and its low cost of production; poor bioavailability remains the major limit to be overcome. Accordingly, Di Giacomo et al. [32] examined the possible neuroprotective role of ferulic acid, a phenolic acid that occurs widely in nature, especially in fruits, vegetables, and cereals, in Alzheimer’s disease (AD). In addition to its ability to prevent neurodegeneration in several brain regions and to inhibit Aβ oligomer aggregations, ferulic acid has also been found to be endowed with antidiabetic properties which have been associated with cognitive benefits produced in an animal model of non-obese type 2 diabetic animals with memory deficits [32]. This evidence suggests a possible interest in ferulic acid as a neuroprotective strategy and underpins future preclinical and clinical studies aimed at unraveling the role of this phenolic acid in the prevention and/or treatment of AD and type 3 diabetes.

Overall, the collected studies enhance our knowledge about the health benefits and nutraceutical potential of plant polyphenols while also prompting further research in crucial areas, such as methods of their extraction from natural sources, enhancing their bioavailability, and exploring their clinical efficacy.
